# Professional Letter

**Published:** 1855-05

**Authors:** 

**Affiliations:** Ohio


### A PROFESSIONAL LETTER, WITH REPORT OF A CASE.

BY EPSOM SAULTZ, JR., M. D.

MESSRS. EDITORS: I cannot better respond to your flattering invitation to contribute to your, to me invaluable Journal, than by giving you a running outline of a case which I had under my care for more than two years, and which has presented some points that to me seemed interesting. I have only to premise that this is my first medical article, and that with you at least, I trust my inexperience will be a sufficient excuse for any want of accuracy in description.

Mr. J. M. C., a retired sea-captain, was of about 65 years of age, of plethoric habit, who had, from early boyhood, been accustomed to the generous diet and stimulating drinks of wealthy sea-captains, and had always enjoyed robust vigorous health. Three years ago, when I moved to this city, I heard remarkable accounts of the wonderful and incomprehensible paroxysms to which Capt. C. was subject, twice or three times every week. Being a young Dr., these accounts of course interested me; but I could learn no facts which gave my professional curiosity any clue to the real nature of the case. He was then under the care, as they charitably called it, of a distinguished Homœopathic Dr., whose diagnosis of disease, as the sequel will show, was generally as mysterious as his treatment. After a year's residence here, during which time I served the usual apprenticeship of all young Drs., and was judiciously held in probation by the discriminating public, I began to earn for myself a title to respectability and confidence, and was gradually allowed to enter some of the families of the wealthy, with that gracious condescention for which I trust I shall ever be truly grateful.

In respect to Capt. C., it seems that two or three years had already been spent amid frequent paroxysms, and the white sugar treatment not having produced, in all this time the desired result, the family were so heretic as to look about for other assistance. Accordingly, late one night, when the suffering man was in one of his attacks, I was graciously sent for; more, as I was at the time informed, for the privilege of witnessing a medical curiosity, than with the hope that anything could be done to relieve him; for his rejected saccharine adviser had of course told him that if *he* could not assist him, nobody could.

As I entered, the phenomena presented were prominent enough. Supported in his chair sat the sufferer; his face of a fearful turgidity, and about the color of dark mahogany; unable to speak, except in fragmentary words, from the incessant rattling, choking coughing expectoration; respiration fearfully hurried and gasping; each effort at inspiration seemingly unsuccessful and apparently the last; expectoration profuse, almost constant, and amounting, at the close of the attack, to half a large wash-bowl full; the fluid expectorated of precisely the color of very red urine, or of port wine, covered with a very tenacious foam. The pulse was very full and hard; a drenching perspiration or rather transpiration covered the upper portion of the body; and the sufferer was indulging in such vivid exclamations for aid as imported an intense sense of suffocation. All these phenomena presented themselves to the eye. On approaching the patient, I could hear at some little distance the rattling bronchial and pulmonary crepitus; and auscultation revealed such astounding crepitus, as I have never heard, before or since. I may add, that the duration of each attack, was about one hour in its greatest severity.

After a protracted and silent examination on my part, the first words uttered by the intelligent wife, were an enquiry as to the correct name and a reasonable explanation of the mysterious disease. In such a case, I felt warranted in going into a clear but simple explanation of the case. Your friend, Epsom Saultz, then proceeded to state, that the disease was plainly enough what is properly called Humoral Asthma, whereupon he was suddenly enlightened by the illuminating intelligence that his pellet predecessor had baptised the disease nothing less, than—Rheumatism of the Stomach! Put a pin there, on homœopathic intelligence.

But to proceed: The patient was entrusted to my efforts, in the hope of a recovery, or at least, of some relief. He had been suffering thus for some two years, and the continuance of the attacks had worn sadly upon his health, and he was compelled to sleep in his chair at night, from the effect of the recumbent posture in inducing the paroxysms. Around this prominent disease, or symptom, asthma, there centered a variety of other affections; indigestion, with alternate constipation and diarrhœa; excessive tympanites; neuralgia; wandering rheumatic pains; dysuria, with symptoms of renal affection; and some suspicion of an affection of the heart.

Being young and sanguine, I confess I was not unwilling to take hold of this case, firmly impressed as I was with the conviction that something *ought* to be done, and might be, rather than with the vanity that I was just the doctor to do it. Of course, the prominent object which I at first set myself to accomplish, was to put a stop, if safely possible, to these fearful paroxysms, which had already seriously impaired his health and vigor, and seemed likely if allowed to continue, to prove indirectly fatal. But how was this object to be accomplished? Here was a field entirely new to me. I asked myself a thousand questions as to the pathological phenomena concerned in the production of the disease, but the obscurity of the cause baffled all my efforts, at satisfactory conclusions. Could the affection date from any lurking inflammatory tendency, or was the tide-like congestion which surged into the lungs at the attacks, due to any excess of the circulating fluid? If either of these, bleeding might be of service; so I tried it; but it did no good. So I was constrained to take another track.

Was it possible that the accumulation in the lungs, constituting a paroxysm, was due to any irritation, which lit up the train of morbid phenomena? If so, sedative treatment of various kinds, and variously employed, might promote my object. I employed them, but the dreaded paroxysms still kept their course.

Perhaps, I said to myself, there may after all be some benefit derived from the employment of anti-spasmodics, for who knows but there may be a purely nervous cause at the bottom of all this trouble? So stramonium was smoked and the round of appropriate treatment was again employed, but the attacks still recurred.

But again, I said, I have not employed any counter irritants, so I will blister; and his feet I find are always cold before an attack; I will attend to that, also, with fomentations. But again and again I was baffled.

I remember well, at this period of the treatment, after I had been chagrined by the trials of some weeks, and my incipient ardor had quite evaporated, that I spent nearly a whole night in thinking and studying over this case, resolved if possible, to come to some more satisfactory conclusion. I had been summoned to his chamber in the evening, and had been a busy, but painfully useless attendant, upon his intense sufferings, and I was stung by my obvious want of success.

A man, I said finally, who loses three or four quarts of bloody serum every week, by this bronchial transudation, must, from that fact alone, demand some constitutional treatment; now I have it, I thought; some form of alteratives and tonics will certainly do it. The next morning I prepared one of the most elaborate combinations of the kind, ever put together; and if the prayers of an anxious doctor do any good, my patient should have been cured by that mixture. But—he was n't.

At length, I made a desperate effort to divest myself of every memory and prejudice in the case; to go back again to the beginning, as if it were a new case and start *de novo*. I analyzed once more, the phenomena concerned in the production of the paroxysms, I criticised every symptom that transpired within half an hour before an attack; I studied all the manifestations of the invasion, to learn if possible what was the subtle cause, and just where and how I should strike the chain of morbid influences that induced an attack, so as to cut it short.

The patient, though even at this time robust and even plethoric, had yet, to my mind, some evidences of constitutional debility, and I could half trace a development of this, in the morbid pathway to an attack. Perhaps this was due to the continued action of the collateral affections, above enumerated; but I had, as far as possible, obviated these and thereby removed every possible obstacle to recovery; but without success. But, possibly this local debility, which may be an indirect cause or occasion of an attack of his asthma, and which may be the particular link in the chain, at which I should strike, possibly it may be primary, as far as I am concerned, and that my efforts should therefore be directed especially to that. I thought, on this view of it, that I could discover some evidences of what I may call a local nervous debility, but just in what shape I could not decide. Might there not be a want of tonicity in the peripheral portion of the nervous structure that supplied the pulmonary vesicles; or, more generally, a partial paralysis of the great sympathetic? I will act on this not unreasonable presumption. My best course I thought would be the administration of some powerful stimulant, in decided and repeated doses, at the earliest moment of warning of a coming paroxysm; by this means I hoped I might rouse the flagging nervous system to a sense of its duty, brace up and fortify the yielding pulmonary system against the threatened serous invasion, and thus enable my patient to stave off an attack. I had heretofore attempted to relive the patient before an attack, of a flatulent accumulation, which always escaped in eructations afterwards, as the crisis subsided, and very much to his relief. I would combine both these designs. I therefore, with a trembling hand, but a hopeful heart, made the following combination:

℞ Æther Sulph.

Tinct. Assafœt.

Aq. Menth. Pip.

Each, equal parts. I lost no time in conveying my new instructions to the patient, with directions to take a table-spoonful of the above at the earliest possible warning of an approaching attack, and a tea-spoonful every five minutes after, till he had either come off victorious, or till he should find it too late to hope for success. He could generally predict an attack from twenty minutes to half an hour, before it fairly invaded him. In two or three days he had an opportunity for its use. It succeeded! When he felt the approach, he summoned his bottle, and poured down the suffocating fluid with a hearty good will, and the first dose gave him assurance of its good effects; it warmed his stomach; it promoted immediate eructation of flatus; it loosed, he said, all those inward bands and cords which prevented his breathing; it produced a sense of glowing warmth and freedom quite delightful. A second dose seemed, according to his description, to open his lungs, and to free him from all restraint. Three or four doses effectually staved off the dreaded invasion, and when I arrived, I found him in jolly humor, delighted at my discovery, with a full good pulse, equable circulation and absence of pulmonary congestion.

To shorten my already too long letter, I will only add that, for nearly two years afterwards, he defended himself from numberless invasions, by this means; and what was better, he found that the use of it, say twice a week for a month or two, to drive off threatened attacks, so modified his system, that for some months after, he had no symptoms of an invasion. At times, I modified the medicine, by substituting Aqua Ammonia for the Ether, and by some other changes; but I generally found my first hit was the best.

The patient survived some two years after we first succeeded in driving off his paroxysms of asthma, and with almost entire exemption from that difficulty. He improved so much as to take a journey to Europe, where he spent a summer on the continent; his bottle of friendly aid always being a faithful pocket companion; and never till the day of his death would he stir without it.

He finally died, from a combination of other and complicated affections, which I have not now room to enumerate. In the later stages of his illness we had so great a variety of morbid conditions to combat, that the old asthma was quite lost sight of. The treatment of this case through all its varying phases, for nearly three years, forcibly impressed me with the fact that the judicious physician must often change the land marks of his treatment, and start all over again, so to speak, upon a new and fresh course. In a chronic case, the present trouble may be asthma, next spring rheumatism, next fall bronchitis, and another year a still fresh form of the same fundamental cause of disease.

An autopsy revealed, as we had expected, Bright's kidney, the result undoubtedly of forty or fifty years of "moderate drinking," and also disease of the mitral valves, the consequence probably of those sweeping tides that were wont to come rushing and crowding into his lungs.

OHIO, March, 1855.

